# Identification of Mutations in the *PYRIN*-Containing *NLR* Genes (*NLRP*) in Head and Neck Squamous Cell Carcinoma

**DOI:** 10.1371/journal.pone.0085619

**Published:** 2014-01-21

**Authors:** Yu Lei, Vivian W. Y. Lui, Jennifer R. Grandis, Ann Marie Egloff

**Affiliations:** 1 Department of Diagnostic Sciences, University of Pittsburgh School of Dental Medicine, Pittsburgh, Pennsylvania, United States of America; 2 Department of Otolaryngology, University of Pittsburgh School of Medicine and University of Pittsburgh Cancer Institute, Pittsburgh, Pennsylvania, United States of America; 3 Department of Pharmacology and Chemical Biology, University of Pittsburgh School of Medicine and University of Pittsburgh Cancer Institute, Pittsburgh, Pennsylvania, United States of America; 4 Department of Microbiology and Molecular Genetics, University of Pittsburgh School of Medicine and University of Pittsburgh Cancer Institute, Pittsburgh, Pennsylvania, United States of America; Barts & The London School of Medicine and Dentistry, Queen Mary University of London, United Kingdom

## Abstract

Head and Neck Squamous Cell Carcinoma (HNSCC) encompasses malignancies that arise in the mucosa of the upper aerodigestive tract. Recent high throughput DNA sequencing revealed HNSCC genes mutations that contribute to several cancer cell characteristics, including dysregulation of cell proliferation and death, intracellular proinflammatory signaling, and autophagy. The PYRIN-domain containing NLR (Nucleotide-binding domain, Leucine rich Repeats – containing) proteins have recently emerged as pivotal modulators of cell death, autophagy, inflammation, and metabolism. Their close physiologic association with cancer development prompted us to determine whether mutations within the *NLRP* (*PYRIN*-containing *NLR*) gene family were associated with HNSCC genome instability and their clinicopathologic correlations. Catastrophic mutational events underlie cancer cell genome instability and mark a point-of-no-return in cancer cell development and generation of heterogeneity. The mutation profiles of 62 patients with primary conventional type HNSCC excluding other histologic variants were analyzed. Associations were tested using Fisher's Exact test or Mann-Whitney U test. Mutations in *NLRP* were associated with elevated genome instability as characterized by higher mutation rates. Clinically, *NLRP* mutations were more frequently found in HNSCC arising in the floor of mouth (50.0%) in comparison with HNSCC at other head and neck locations (14.8%). These mutations were clustered at the leucine rich repeats region of NLRP proteins, and affected *NLRP* genes were mostly localized at chromosomes 11p15.4 and 19q13.42-19q13.43. Twenty novel *NLRP* mutations were identified in HNSCC, and mutations in this group of genes were correlated with increased cancer cell genome mutation rates, and such features could be a potential molecular biomarker of HNSCC genome instability.

## Introduction

Recent technological advances in whole exome sequencing at much greater depths provide us with an unparalleled opportunity to interrogate the human cancer genome for mutational profiles. Studies on the evolutionary history of cancer genomes reveal that catastrophic mutational events (also referred to as “kataegis”, a Greek word meaning thunderstorm or shower), which are characterized by rapid accumulation of point mutations at clustered regions, may mark a “point-of-no-return” during cancer development by giving rise to subclones of cancer cells [1], [2]. Genome instability exemplified by kataegis and chromothripsis represents a hallmark of cancer [3].

Head and Neck Squamous Cell Carcinoma (HNSCC) encompasses malignancies that arise in the mucosa of the upper aerodigestive tract, accounting for 300,000 annual deaths and ranking 6^th^ among the most common human cancers [4]. Due to the characteristic anatomic location, the upper aerodigestive pathway, especially the oral cavity, is constantly exposed to environmental factors, many of which possess potent carcinogenic capacity. Indeed, the geographic variation of HNSCC incidence corresponds well with the exposure to risk factors including tobacco use and human papilloma virus (HPV) infection [5]. The unique environmental etiologic factors in HNSCC development suggest the functional significance of genes involved in host-environment and/or host-pathogen interactions. Novel mutations in genes regulating squamous epithelial differentiation are unveiled in recent endeavors to map HNSCC mutational landscape [6], [7]. However, the genetic signatures that may reflect the overall genome instability in HNSCC are yet to be determined. This study aims to explore the significance of mutations in a group of genes modulating host-environment interactions and their clinicopathologic correlations.

Emerging evidence place a novel gene family at the forefront of host-environment interactions. *NLR* (nucleotide-binding, lots of leucine-rich repeats containing) gene family (initially coined as *CATERPILLER*, also known as *NOD* and *NALP*) is characterized by a central nucleotide-binding domain, a C-terminal leucine rich repeats (LRR) domain, and an N-terminal effector domain. The N-terminus could be CARD, PYRIN, BIR, AD or X domain, which shows certain homology with CARD or PYRIN yet cannot be categorized into either [8]. By engaging with the formation of distinct protein complexes, NLR proteins play central roles in modulating host responses to both PAMPs (pathogen-associated molecular patterns) and DAMPs (damage-associated molecular patterns) [9], [10].

A variety of NLR-mediated signaling pathways convey meticulous regulatory mechanisms in cell death [11], inflammation [9], [10], [12], autophagy [13]–[16], and more recently carcinogenesis [17]–[24]. Among the NLR family, a subgroup of NLRs harboring an N-terminal PYRIN domain have been implicated in malignancies arising in the lower digestive tract. Indeed, genetic deficiency in *Nlrp3* results in increased colitis-associated colon cancer [19], [25]. NLRP6 controls colonic microbial ecology and colorectal epithelial cells renewal; and its deficiency results in increased colitis and colorectal cancer [21]–[23]. *NLRP7* variants are involved in post-molar choriocarcinoma, and a germline mutation of *NLRP2* is associated with a proliferation disorder known as Beckwith-Wiedemann syndrome [26], [27].

A significant portion of HNSCC is comprised of cancer of the oral cavity, which is not only directly exposed to a variety of PAMPs and DAMPs, but also constantly inhabited by a microbiota composed of more than 700 bacterial species [28]. Alteration of oral microbiota as seen in those with chronic adult periodontitis or poor oral hygiene has been correlated with the development of several types of cancer [29], [30]. Given the significance of NLR proteins in host inflammatory responses, autophagy, normal epithelial renewal, and emerging roles in maintaining microbiota homeostasis [13], [18], [21]–[23], their deficiency may change host responses to environmental insults and adjuvant therapeutic agents. However, their specific functions in modulating cancer cell development require further investigations. In this study, we characterize the mutational profiles of 10 *NLRP* genes in 62 primary conventional type HNSCC tumors, and investigate the significance of these mutations in overall cancer genome instability and their correlations with the clinicopathologic characteristics of HNSCC patients.

## Results

### Identification of *NLRP* mutations in HNSCC

A solution-phase hybrid capture and whole exome sequencing with a mean of 150-fold sequence coverage at targeted exonic regions were performed on HNSCC specimens as previously reported [6]. Among all sequenced specimens in the previous study [6], several histologic variants of primary or recurrent HNSCC existed including conventional type SCC (squamous cell carcinoma), basaloid SCC, papillary SCC, spindle cell carcinoma, adenosquamous cell carcinoma, and hybrid verrucous SCC. The latest World Health Organization classification of head and neck tumors (2005) presents a general consensus that these variants may display varied clinical courses and prognoses compared to conventional type SCC. For instance, basaloid squamous cell carcinoma and adenosquamous cell carcinoma are considered to behave more aggressively with early metastasis; papillary squamous cell carcinoma and verrucous carcinoma are slow-growing tumors and may show better prognosis than conventional HNSCC. In order to a perform analyses in a relatively more homogenous population, we restricted our study to 62 patients with primary conventional type HNSCC.

**Figure 1:**
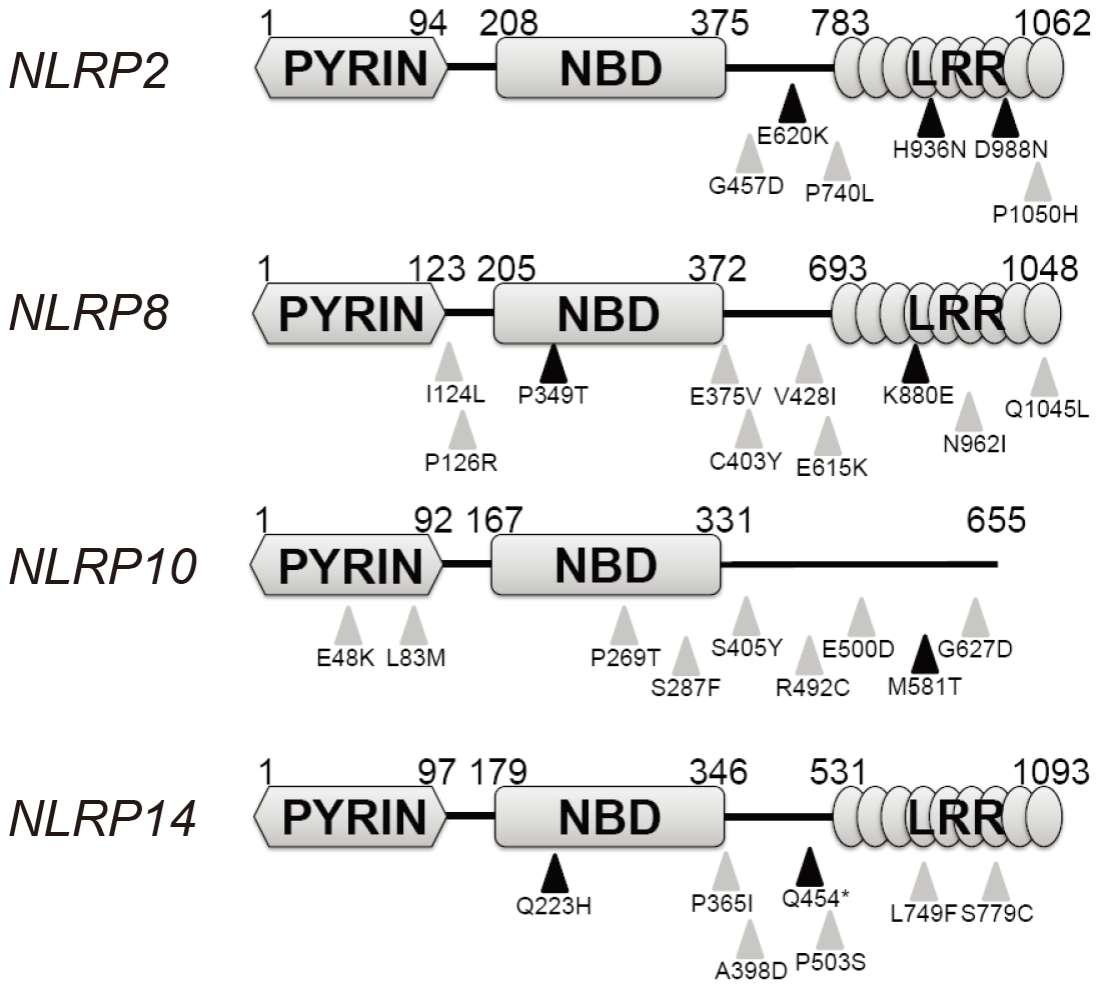
Identification of *NLRP* mutations in FOM HNSCC. Mutations in four *NLRP* genes were identified in FOM HNSCC patients. Black triangles indicate novel mutations identified in this study. Gray triangles represent reported mutations in the Catalogue of Somatic Mutations in Cancer (COSMIC) database.

Non-silent mutations were detected in 10 of 14 human *NLRP* genes (*NLRP1-14*). The whole group of the *NLRP* genes was affected except for *NLRP6*, *NLRP7*, *NLRP9*, and *NLRP13*. Non-silent *NLRP* mutations were present in tumors from 13 patients (Table 1). Mutations in more than 1 *NLRP* genes were identified in 3 tumors. Despite the unknown genetic or environmental predisposition for cancer development at the floor of mouth (FOM), this site is recognized as being a high risk site for HNSCC [31]. Hence, we also reviewed the *NLRP* mutational profiles in 8 FOM HNSCC tumors; four tumors harbored *NLRP* mutations. In addition to our study of 62 conventional HNSCC, *NLRP* mutations have also been reported in HNSCC in an independent whole exome sequencing study of HNSCC [7] and in the Catalog of Somatic Mutations in Cancer (COSMIC) database. *NLRP* mutations most frequently occur at the C-terminus followed by the NBD domain (Figure 1).

**Table 1:** Identification of *NLRP* mutations in primary HNSCC.

Chromosome Location	*NLRP* Genes
**19q13.42-19q13.43**	*NLRP2, NLRP4, NLRP5, NLRP8, NLRP11, NLRP12*
**11p15.4**	*NLRP10, NLRP14*
**17p13.2**	*NLRP1*
**1q44**	*NLRP3*

Novel mutations were identified in 10 *NLRP* genes in HNSCC. Most mutations were involving chromosomal locations 11p15.4 and 19q13.42-19q13.43.

### *NLRP* mutations were associated with increased cancer genome instability

**Figure 2:**
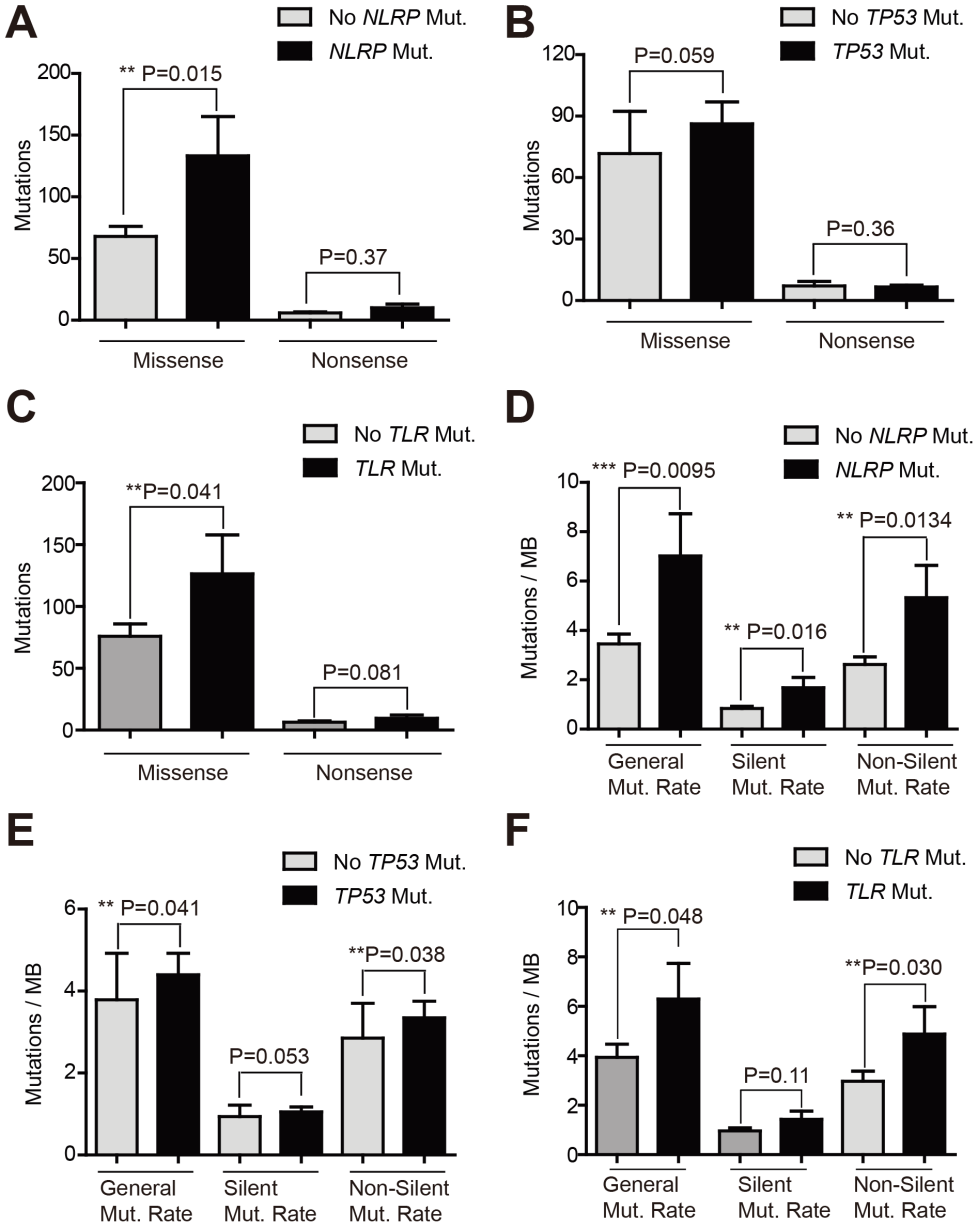
Mutation rates comparisons. (A) Numbers of missense and nonsense mutations were compared between tumors with or without *NLRP* mutations. (B) Numbers of missense and nonsense mutations were compared between tumors with or without *TP53* mutations. (C) Numbers of missense and nonsense mutations were compared between tumors with or without *TLR* genes mutations. (D) Mutation rates [shown as mutations per million base (MB) pairs] were compared between tumors with or without *NLRP* mutations. (E) Mutation rates [shown as mutations per million base (MB) pairs] were compared between tumors with or without *TP53* mutations. (F) Mutation rates [shown as mutations per million base (MB) pairs] were compared between tumors with or without *TLR* genes mutations. P value less than 0.05 was considered significant.

Catastrophic mutational events reflect a high degree of genome instability, which is one of the hallmarks of cancer. Thus we evaluated whether mutations in *NLRP* genes reflected the overall cancer genome instability. Tumors without *NLRP* mutations harbor an average of 68 missense mutations, while those with *NLRP* mutations demonstrate as twice as many missense mutations across their exomes (P = 0.015) (Figure 2A). In agreement with previous findings, two recent large-scale sequencing studies also identified frequent mutations of *TP53* in HNSCC [6], [7], which may drive kataegis in a fraction of the tumors. However, the missense mutation rate in tumors without *TP53* mutations was comparable to that in those with *TP53* mutations (Figure 2B). Despite of the similar size between the human gene family of *TLR*, which is comprised of 10 genes (*TLR1-10*), and pyrin-containing *NLRs*, *TLR* gene mutations were only identified in 7 tumors. Both selected gene members of the *TLR* or *NLRP* families, which were mutated in our cohort of 62 tumors, and total members of both families were compared in light of the length of coding regions. Our analysis showed that the coding regions of both groups of genes were similar (Figure S2). Thus, we employed the *TLR* gene family as another control for our gene mutation analysis. The missense mutations in tumors with *TLR* mutations were higher than those without *TLR* mutations with a marginal P value (P = 0.041) (Figure 2C). In addition, HNSCC with *NLRP* mutations displayed generalized elevated genome instability exemplified by general mutation rate (P = 0.0095), silent mutation rate (P = 0.016), and non-silent mutation rate (P = 0.0134) (Figure 2D). HNSCC with *TP53* mutations also demonstrated higher general mutation rate and non-silent mutation rates (P = 0.041 and P = 0.038, respectively) yet similar silent mutation rates (Figure 2E). Similar to tumors with *TP53* mutations, the tumors with *TLR* mutations more commonly demonstrated higher general mutation rates and non-silent mutation rates (P = 0.048 and P = 0.030, respectively) (Figure 2F). However the silent mutation rates between tumors with or without *TLR* genes mutations were comparable (Figure 2F).

### Demographic information of patients involved in the study

Primary conventional HNSCC has a strong predilection for males; however, this gender predilection appeared to be mitigated in patients harboring *NLRP* mutations, as the representation among females with HNSCC increased from 24% of all included patients in this study to almost 40% among those patients with *NLRP* mutations, although this difference was not statistically significant (Table 2). Neither tobacco nor alcohol use was associated with these mutations. Most examined clinical parameters, such as disease stage, histologic grade, human papilloma virus (HPV) status, 5-year survival and disease progression, did not differ between the patients with and without *NLRP* mutations (Table 2).

**Table 2:** Demographic Profile of Study Subjects with Primary Conventional HNSCC (PC-SCC).

	All PC-SCC Cases		PC-SCC Cases with *NLRP* mutation		PC-SCC Cases without *NLRP* mutation		
	n = 62		n = 13			n = 49	P*
Gender							
Male	47	75.8%	8	61.5%	39	79.6%	0.27∥
Female	15	24.2%	5	38.5%	10	20.4%	
Age, years							
Average (Range)	57 (33–76)		59 (39–75)		56 (33–76)		0.30^§^
Cigarette Pack-Years							
Never smoker	10	16.1%	3	23.1%	7	14.3%	0.62∥
1–49 py	38	61.3%	8	61.5%	30	61.2%	
≥50 py	14	22.6%	2	15.4%	12	24.5%	
Alcohol Quantity†							
Never drinker	12	19.4%	4	30.8%	8	16.3%	0.13∥
1–4	7	11.3%	3	23.1%	4	8.2%	
≥5	41	66.1%	6	46.2%	35	71.4%	
Unknown	2	3.2%	0	0.0%	2	4.1%	
Tumor Site							
Oral Cavity	31	50.0%	8	61.5%	23	46.9%	0.98∥
Oropharynx	10	16.1%	2	15.4%	8	16.3%	
Hypopharynx	8	12.9%	1	7.7%	7	14.3%	
Larynx	11	17.7%	2	15.4%	9	18.4%	
Sinonasal	2	3.2%	0	0.0%	2	4.1%	
Disease Stage							
I	0	0.0%	0	0.0%	0	0.0%	0.87∥
II	5	1.4%	1	7.7%	4	8.2%	
III	12	3.4%	3	23.1%	9	18.4%	
IV	45	12.7%	9	69.2%	36	73.5%	
Histologic Grade							
Well Differentiated	1	1.6%	0	0.0%	1	2.0%	0.80∥
Moderately Differentiated	39	62.9%	9	69.2%	30	61.2%	
Poorly Differentiated	22	35.5%	4	30.8%	18	36.7%	
HPV Status							
Positive	10	16.1%	1	7.7%	9	18.4%	0.67∥
Negative	52	83.9%	12	92.3%	40	81.6%	
Treatment							
RT Only	11	17.7%	4	30.8%	7	14.3%	0.29∥
CRT	38	61.3%	6	46.2%	32	65.3%	
No CRT	13	21.0%	3	23.1%	10	20.4%	
Unknown	0	0.0%	0	0.0%	0	0.0%	
Vital Status							
Alive	29	46.8%	6	46.2%	23	46.9%	1.00∥
Dead	33	53.2%	7	53.8%	26	53.1%	
5-year overall survival, months							
Median (Range)	34.3 (2.0–60.0)		22.1 (2.4–58.0)		37.9 (4.6–60.0)		0.58¶
Disease Progression							
No disease progression	22	35.5%	6	46.2%	16	32.7%	0.27∥
Local/Regional Recurrence	12	19.4%	0	0.0%	12	24.5%	
Second Primary	2	3.2%	0	0.0%	2	4.1%	
Distant Metastasis	8	12.9%	2	15.4%	6	12.2%	
Death without Recurrence	18	29.0%	5	38.5%	13	26.5%	
5-year Progression Free Survival, months							
Median (Range)	20.3 (2.0–60.0)		19.1 (2.4–58.0)		21.5 (4.6–60.0)		0.78¶

Contingency tables were analyzed by Fisher's exact test. Survival distributions were analyzed by Log Rank test. Notes:

† Typical number of alcohol drinks in 2 week period, §Mann-Whitney U test,

∥ Fisher's exact test,

¶ Log rank test,

* Comparing patients with or without mutations in *NLRP* genes.

### *NLRP* mutations were associated with HNSCC arising in the floor of mouth (FOM)

**Figure 3:**
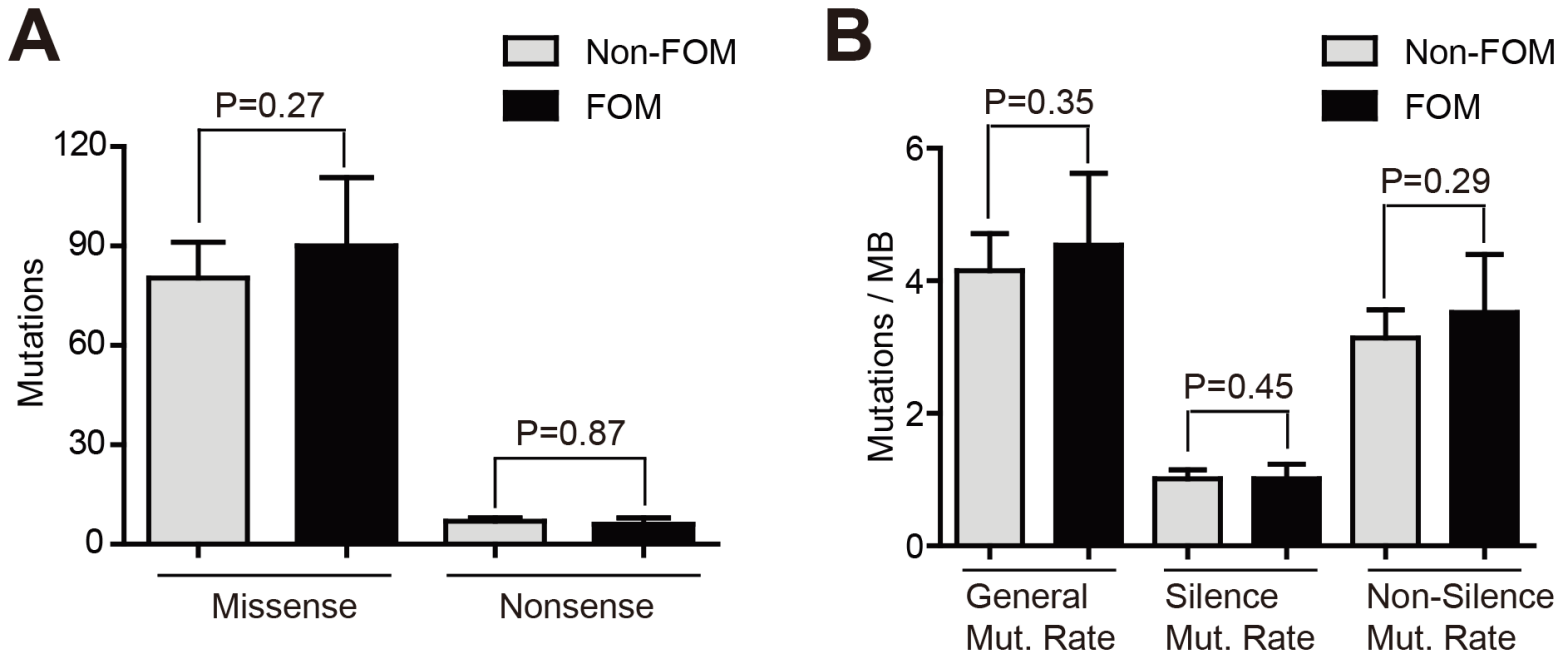
Mutation rates of FOM HNSCC. (A) Numbers of missense and nonsense mutations were compared between patients with FOM or non-FOM HNSCC. (B) Mutation rates were compared between patients with FOM or non-FOM HNSCC.

Floor of mouth is an unequivocal high risk site for HNSCC according to the recent WHO Classification of Head and Neck Tumours (2005). In a comprehensive study of 3,360 specimens, leukoplakia at floor of mouth has the highest incidence showing epithelial dysplasia and carcinoma compared to leukoplakic lesions at all other anatomic locations in the oral cavity [32]. However, the genetic alterations that may occur in this region leading to development of tumors at this anatomical site remain poorly understood. We assessed the correlation between *NLRP* mutations and tumor site in 62 patients. *NLRP* mutations were more common in HNSCC arising in the FOM (P = 0.079 in comparison with HNSCC in other non-FOM oral cavity locations, P = 0.034 in comparison with HNSCC in other non-FOM head and neck locations) (Table 3). To determine whether the clustering of *NLRP* mutations in this site was because of elevated genome instability of FOM HNSCC compared to HNSCC at other sites, we evaluated missense mutation, nonsense mutation, and mutation rates. No significant differences were identified between FOM and non-FOM groups (Figure 3A, 3B), indicating that the higher rate of *NLRP* mutations in FOM HNSCC was not a nonspecific reflection of an overall higher mutation rate at this anatomic site. In order to further substantiate the specificity of this association, we also explored whether mutations in *TP53* or *TLR* genes were clustered in FOM HNSCC. Although mutations of *TP53* or *TLR* genes were seen in a group of patients with higher general mutation rate and non-silent mutation rate, these mutations were not associated with HNSCC arising in FOM (Tables S1 and S2).

**Table 3:** Mutations in *NLRP* genes were more frequently found in FOM HNSCC.

	*NLRP* Mut.	w/o *NLRP* mut.	P value
**FOM**	4	4	
**Non-FOM**	4	19	0.079
**(all other OC locations)**			
**FOM**	4	4	
**Non-FOM**	8	46	0.034
**(all other H&N locations)**			

Contingency table comparisons were made by Fisher's Exact test. P value of less than 0.1 was considered significant.

### Identification of factors affecting the survival of patients with primary conventional type HNSCC

**Figure 4:**
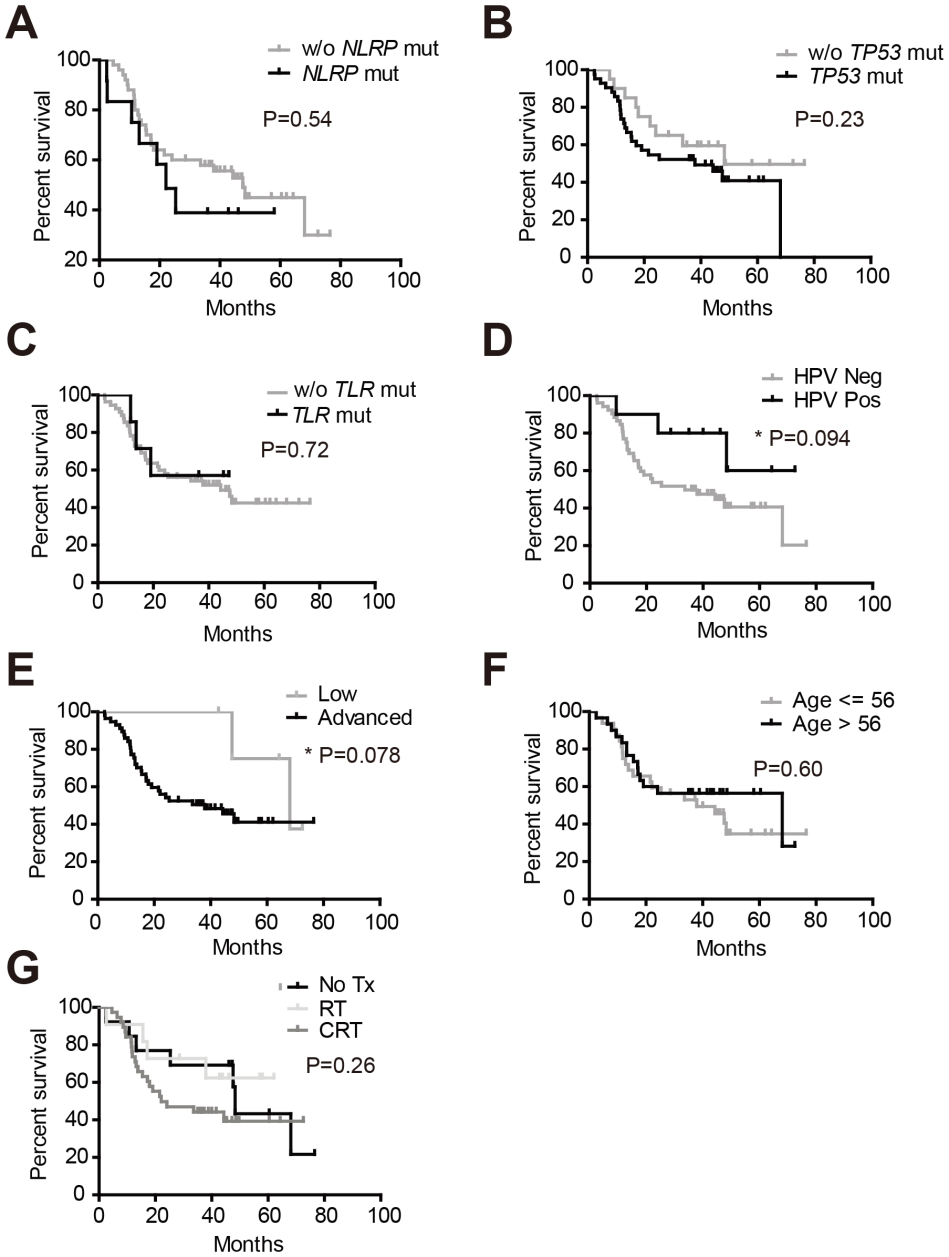
Determination of factors affecting overall survival. Patients overall survival analyses were made between two groups: (A) patients with or without mutations in *NLRP* genes; (B) patients with or without mutations in *TP53* genes; (C) patients with tumors that harbored *TLR* mutations and those without *TLR* mutations; (D) patients with or without HPV infections; (E) patients with low stage (stage II) or advanced stage (stage III and beyond) tumors; (F) patients of less or more than 56 years old; (G) patients who received no adjuvant therapy, radiotherapy alone or both chemotherapy and radiotherapy.

Tumors harboring *NLRP* mutations had significantly increased general mutation rate and missense mutations; however, the presence of these mutations was not associated with patients overall survival (Figure 4A). Although *TP53* mutations were present in 67.7% of the total tumor specimens, these mutations did not affect patient survival (Figure 4B). The presence of *TLR* mutations had little prognostic value in patients survival (Figure 4C). Among all factors tested, only HPV infection status and tumor stage were associated with overall survival. In agreement with previous literatures [33], [34], patients with HPV-positive tumors had improved survival (P = 0.094) (Figure 4D). In fact, HPV-positive tumors demonstrated a significantly lower missense mutations and general mutation rates (Figure S1A, S1B). Despite the fact that the majority of the patients involved in this study were at advanced stage (stage III and beyond), our analysis showed low stage status positively impacted overall survival (Figure 4E). However, tumor stage was not associated with increased genome instability (Figure S1C, S1D). Neither age nor adjuvant therapies such as chemotherapy or radiotherapy were associated with patients survival (Figure 4F–G).

## Discussion

NLRP proteins are evolutionarily and functionally conserved. The *NLR* gene family was initially discovered through genomic database mining based on structural homology [9]. By incorporating an N-terminal effector domain, a central nucleotide-binding domain, and a variable number of LRR at the C-terminus, the 22 human NLR proteins are structurally similar to the plant R protein, which conveys resistance to pathogens [8]. The pioneering research on NLR proteins primarily focused on their pivotal roles in modulating inflammatory responses such as caspase-1 activation, MAPK, NF-κB, and mitochondria-based antiviral signaling [9], [10], [13], [35]. Several NLRs possess similar functions in facilitating the assembly of a large multimeric protein complex, coined as the inflammasome, to process pro-caspase-1 into its mature form, which induces the maturation and secretion of IL-1β and IL-18, in response to a variety of PAMPs and DAMPs [9]. Both HNSCC derived cell lines and invasive HNSCC tumor cells produce proinflammatory cytokines including IL-1β, TNF-α, and IL-6 [36], [37]. In addition, elevated salivary IL-1β has been found in oral squamous cell carcinoma patients [38]. Among the NLRP proteins, NLRP1, 2, 3, and 12 participate in the formation and activation of inflammasome [9], and mutations in these genes were identified in HNSCC.

HNSCC is notorious for its heterogeneity and frequent resistance to adjuvant therapies. In addition to the aforementioned inflammatory pathways, NLR proteins have also been implicated in the regulation of autophagy, which conveys resistance to a variety of adjuvant therapeutic agents [39]. A NLR protein NOD2 induces autophagy in dendritic cells upon engagement with muramyldipeptide [14]. NLRP4 associates with beclin1 to negatively regulate autophagy [15]. NLRX1 modulates autophagy by recruiting ATG12, ATG5, and ATG16L1 to a large mitochondrial protein complex, through an intermediary partner TUFM [13], [16]. Inhibition of autophagy is proven an effective strategy in sensitizing HNSCC cells to a number of adjuvant therapeutic agents. The majority of the mutation of *NLRP* genes in HNSCC are located at the C-terminal LRR domain. With the roles of NLR proteins in modulating autophagy being unveiled, it would be necessary to evaluate the function of these autophagy-related NLRs in modulating cancer cell resistance to novel adjuvant therapy.

One critical event that precedes the generation of cancer cell heterogeneity is kataegis, in which rapid mutations accumulate in “hotspots” to drive the generation of subclones of cancer cells [1], [2]. Although mutations in tumor suppressor genes such as *TP53* are unequivocally involved in many HNSCC, they do not necessarily define the idiosyncratic genetic features of an individual tumor. In fact, we noted that the missense mutations were comparable between patient tumors with or without *TP53* mutations. It is likely specific rarer mutational events in a subset of genes that shape the biologic features of an individual tumor, such as capability of forming subclones. These subclones may contribute to divergent responses to adjuvant treatments. It is possible that specific anatomical sites may have a propensity to develop tumors with mutations in a specific set of genes. Compared to keratinized mucosa lining the gingiva, buccal mucosa, and hard palate, mucosa lining the floor of mouth is non-keratinized, which makes this site more prone to environmental insults. Our findings that a group of genes pivotal in modulating host-environment insults interactions were frequently mutated in HNSCC arising in the floor of mouth suggest their functional significance in cancer development. Indeed, we found that mutations in *NLRP* genes were closely associated with higher degree of cancer genome instability. The small number of primary FOM HNSCC analyzed in this study is a limitation. However, the 62 tumors analyzed represented the common anatomic sites of primary HNSCC. In addition, we employed mutations of the *TP53* gene and the *TLR* gene family as specificity controls. Of the genes analyzed, the association with FOM was unique to the mutations of the *NLRP* genes.

In agreement with previous studies [33], [34], we found HPV status and tumor stage were associated with HNSCC patients overall survival. Although genome instability exemplified by kataegis represents a defining step in driving the diversity of tumor subclones, it did not appear to be a reliable prognostic factor. For example, while HPV negative HNSCC patients had a significantly elevated level of general mutation rate, advanced stage tumors did not necessarily display worse genome instability (Figure S1). However, increased intra-tumor heterogeneity resulting from non-driver mutations in a kataegis event may substantially affect the tumor adaptation to treatment, including evolving resistance to therapy [40]. Hence, the rarer mutations especially those reflecting genome instability may not be stochastic, rather they may be the result of a collective response to PAMP/DAMP challenge and adaptive response to therapeutic inflictions, contributing to the establishment of resistance.

In Conclusion, We Further Characterized The Genetic And Clinicopathologic Profile Of The Novel *Pyrin*-Containing *Nlr* Gene Family In 62 Patients With Conventional Type Hnscc. Clinically, These Mutations Were Frequently Found In Hnscc Arising In The Floor Of Mouth. These Mutations Were Clustered At The Lrr Domain Of Nlrp Proteins; And The Affected *Nlrp* Genes Were Mostly Localized At Chromosomes 11p15.4 And 19q13.42-19q13.43. Mutations In The *Nlrp* Genes Were Associated With Enhanced Genome Instability In Hnscc.

## Materials and Methods

### Ethics statement

All clinical data including patients' demographic information, tumor histologic type and grading, genetic mutation identity, adjuvant treatment information, and vital status were made available through the Specialized Program of Research Excellence (SPORE) in Head and Neck Cancer neoplasm of the University of Pittsburgh. Patients included in this study were enrolled into the Head and Neck tumor bank protocol. This protocol requires written consent and was approved by the Institutional Review Board of the University of Pittsburgh.

### Study subjects

Whole exome sequencing was performed on 62 patients with primary or recurrent HNSCC as previously described [6]. Only patients with primary conventional type squamous cell carcinoma were included. Recurrent tumors or other histologic variants, such as basaloid squamous cell carcinoma, papillary squamous cell carcinoma, spindle cell carcinoma, adenosquamous cell carcinoma, and hybrid verrucous squamous cell carcinoma, were excluded. All participating patients were Caucasians, and other demographic information was summarized in Table 2.

### Data Deposition

Identified mutations associated with our recent whole exome sequencing effort were made available in dbGaP with the accession # phs000370.v1.p1 as previously described [6]. All novel mutations were also available through the COSMIC database. In order to differentiate the novel mutations associated with tumors analyzed in this study and those that had been present in the COSMIC database, we highlighted all novel mutations with a black triangle in Figure 1.

### Gene family coding region calculations

The numbers of amino acids of each member of the TLR or NLRP families were retrieved from the National Center for Biotechnology Information (NCBI) protein database, and the lengths of the coding regions were determined by the number of the amino acids multiplied by three. The comparison was analyzed by Mann-Whitney U test, and a P value of less than 0.05 was considered significant.

### Statistical Analyses

Comparisons of mutation rates and average ages between the two groups were made by Mann-Whitney U test. Fisher's exact test was employed to analyze contingency tables. Survival distributions were analyzed by Log Rank test. Analyses were made using Graphpad Prism 5.0 (Graphpad Software, Inc.). P value of less than 0.1 was considered to be significant.

## Supporting Information

Figure S1 **Mutation rates comparisons.** (A) Numbers of missense and nonsense mutations were compared between patients with or without HPV infection. (B) Mutation rates were compared between patients with or without HPV infection. (C) Numbers of missense and nonsense mutations were compared between patients with low stage or advanced stage SCC. (D) Mutation rates were compared between patients with low stage or advanced stage SCC. P value less than 0.05 was considered significant.
(TIF)

Figure S2 **Coding region lengths comparisons.** (A) The coding region lengths between selected members of the *TLR* and *NLRP* gene families, which were mutated in our cohort, were compared by Mann-Whitney U test. (B) The coding region lengths between the total members of the *TLR* and *NLRP* gene families were compared by Mann-Whitney U test. P value of less than 0.05 was considered significant.
(TIF)

Table S1 **Mutations of the** ***TP53*** **gene were not enriched in FOM HNSCC.** Contingency table comparisons were made by Fisher's Exact test to investigate whether mutations of the *TP53* gene were more frequently seen in HNSCC arising FOM. P value of less than 0.1 was considered significant.
(DOCX)

Table S2 **Mutations of the** ***TLR*** **genes were not enriched in FOM HNSCC.** Contingency table comparisons were made by Fisher's Exact test to investigate whether mutations of the *TLR* gene family were more frequently seen in HNSCC arising FOM. P value of less than 0.1 was considered significant.
(DOCX)

## References

[1] Nik-Zainal S, Alexandrov LB, Wedge DC, Van Loo P, Greenman CD, et al (2012) Mutational processes molding the genomes of 21 breast cancers. Cell 149: 979–993. 22608084 10.1016/j.cell.2012.04.024 PMC3414841

[2] Nik-Zainal S, Van Loo P, Wedge DC, Alexandrov LB, Greenman CD, et al (2012) The life history of 21 breast cancers. Cell 149: 994–1007. 22608083 10.1016/j.cell.2012.04.023 PMC3428864

[3] Hanahan D, Weinberg RA (2011) Hallmarks of cancer: the next generation. Cell 144: 646–674. 21376230 10.1016/j.cell.2011.02.013

[4] Ferlay J, Shin HR, Bray F, Forman D, Mathers C, et al (2010) Estimates of worldwide burden of cancer in 2008: GLOBOCAN 2008. Int J Cancer 127: 2893–2917. 21351269 10.1002/ijc.25516

[5] van Monsjou HS, Balm AJ, van den Brekel MM, Wreesmann VB (2010) Oropharyngeal squamous cell carcinoma: a unique disease on the rise? Oral Oncol 46: 780–785. 20920878 10.1016/j.oraloncology.2010.08.011

[6] Stransky N, Egloff AM, Tward AD, Kostic AD, Cibulskis K, et al (2011) The mutational landscape of head and neck squamous cell carcinoma. Science 333: 1157–1160. 21798893 10.1126/science.1208130 PMC3415217

[7] Agrawal N, Frederick MJ, Pickering CR, Bettegowda C, Chang K, et al (2011) Exome sequencing of head and neck squamous cell carcinoma reveals inactivating mutations in NOTCH1. Science 333: 1154–1157. 21798897 10.1126/science.1206923 PMC3162986

[8] Ting JP, Lovering RC, Alnemri ES, Bertin J, Boss JM, et al (2008) The NLR gene family: a standard nomenclature. Immunity 28: 285–287. 18341998 10.1016/j.immuni.2008.02.005 PMC2630772

[9] Davis BK, Wen H, Ting JP (2011) The Inflammasome NLRs in Immunity, Inflammation, and Associated Diseases. Annu Rev Immunol 10.1146/annurev-immunol-031210-101405 PMC4067317 21219188

[10] Ting JP, Duncan JA, Lei Y (2010) How the noninflammasome NLRs function in the innate immune system. Science 327: 286–290. 20075243 10.1126/science.1184004 PMC3943909

[11] Ting JP, Willingham SB, Bergstralh DT (2008) NLRs at the intersection of cell death and immunity. Nat Rev Immunol 8: 372–379. 18362948 10.1038/nri2296

[12] Lamkanfi M, Dixit VM (2009) Inflammasomes: guardians of cytosolic sanctity. Immunol Rev 227: 95–105. 19120479 10.1111/j.1600-065X.2008.00730.x

[13] Lei Y, Wen H, Yu Y, Taxman DJ, Zhang L, et al (2012) The mitochondrial proteins NLRX1 and TUFM form a complex that regulates type I interferon and autophagy. Immunity 36: 933–946. 22749352 10.1016/j.immuni.2012.03.025 PMC3397828

[14] Cooney R, Baker J, Brain O, Danis B, Pichulik T, et al (2010) NOD2 stimulation induces autophagy in dendritic cells influencing bacterial handling and antigen presentation. Nat Med 16: 90–97. 19966812 10.1038/nm.2069

[15] Jounai N, Kobiyama K, Shiina M, Ogata K, Ishii KJ, et al (2011) NLRP4 negatively regulates autophagic processes through an association with beclin1. J Immunol 186: 1646–1655. 21209283 10.4049/jimmunol.1001654

[16] Lei Y, Wen H, Ting JP (2013) The NLR protein, NLRX1, and its partner, TUFM, reduce type I interferon, and enhance autophagy. Autophagy 9: 432–433. 23321557 10.4161/auto.23026 PMC3590269

[17] Zaki MH, Vogel P, Malireddi RK, Body-Malapel M, Anand PK, et al (2011) The NOD-like receptor NLRP12 attenuates colon inflammation and tumorigenesis. Cancer Cell 20: 649–660. 22094258 10.1016/j.ccr.2011.10.022 PMC3761879

[18] Allen IC, Wilson JE, Schneider M, Lich JD, Roberts RA, et al (2012) NLRP12 suppresses colon inflammation and tumorigenesis through the negative regulation of noncanonical NF-kappaB signaling. Immunity 36: 742–754. 22503542 10.1016/j.immuni.2012.03.012 PMC3658309

[19] Allen IC, TeKippe EM, Woodford RM, Uronis JM, Holl EK, et al (2010) The NLRP3 inflammasome functions as a negative regulator of tumorigenesis during colitis-associated cancer. J Exp Med 207: 1045–1056. 20385749 10.1084/jem.20100050 PMC2867287

[20] Zaki MH, Lamkanfi M, Kanneganti TD (2011) The Nlrp3 inflammasome: contributions to intestinal homeostasis. Trends Immunol 32: 171–179. 21388882 10.1016/j.it.2011.02.002 PMC3070791

[21] Chen GY, Liu M, Wang F, Bertin J, Nunez G (2011) A functional role for Nlrp6 in intestinal inflammation and tumorigenesis. J Immunol 186: 7187–7194. 21543645 10.4049/jimmunol.1100412 PMC3133458

[22] Elinav E, Strowig T, Kau AL, Henao-Mejia J, Thaiss CA, et al (2011) NLRP6 inflammasome regulates colonic microbial ecology and risk for colitis. Cell 145: 745–757. 21565393 10.1016/j.cell.2011.04.022 PMC3140910

[23] Normand S, Delanoye-Crespin A, Bressenot A, Huot L, Grandjean T, et al (2011) Nod-like receptor pyrin domain-containing protein 6 (NLRP6) controls epithelial self-renewal and colorectal carcinogenesis upon injury. Proc Natl Acad Sci U S A 108: 9601–9606. 21593405 10.1073/pnas.1100981108 PMC3111299

[24] Strowig T, Henao-Mejia J, Elinav E, Flavell R (2012) Inflammasomes in health and disease. Nature 481: 278–286. 22258606 10.1038/nature10759

[25] Zaki MH, Vogel P, Body-Malapel M, Lamkanfi M, Kanneganti TD (2010) IL-18 production downstream of the Nlrp3 inflammasome confers protection against colorectal tumor formation. J Immunol 185: 4912–4920. 20855874 10.4049/jimmunol.1002046 PMC3104023

[26] Meyer E, Lim D, Pasha S, Tee LJ, Rahman F, et al (2009) Germline mutation in NLRP2 (NALP2) in a familial imprinting disorder (Beckwith-Wiedemann Syndrome). PLoS Genet 5: e1000423. 19300480 10.1371/journal.pgen.1000423 PMC2650258

[27] Slim R, Coullin P, Diatta AL, Chebaro W, Courtin D, et al (2012) NLRP7 and the genetics of post-molar choriocarcinomas in Senegal. Mol Hum Reprod 18: 52–56. 21948117 10.1093/molehr/gar060

[28] Ahn J, Chen CY, Hayes RB (2012) Oral microbiome and oral and gastrointestinal cancer risk. Cancer Causes Control 23: 399–404. 22271008 10.1007/s10552-011-9892-7 PMC3767140

[29] Meyer MS, Joshipura K, Giovannucci E, Michaud DS (2008) A review of the relationship between tooth loss, periodontal disease, and cancer. Cancer Causes Control 19: 895–907. 18478344 10.1007/s10552-008-9163-4 PMC2723958

[30] Meurman JH (2010) Oral microbiota and cancer. J Oral Microbiol 2. 10.3402/jom.v2i0.5195 PMC3084564 21523227

[31] Mashberg A, Samit A (1995) Early diagnosis of asymptomatic oral and oropharyngeal squamous cancers. CA Cancer J Clin 45: 328–351. 7583906 10.3322/canjclin.45.6.328

[32] Neville BW, Day TA (2002) Oral cancer and precancerous lesions. CA Cancer J Clin 52: 195–215. 12139232 10.3322/canjclin.52.4.195

[33] Ang KK, Harris J, Wheeler R, Weber R, Rosenthal DI, et al (2010) Human papillomavirus and survival of patients with oropharyngeal cancer. N Engl J Med 363: 24–35. 20530316 10.1056/NEJMoa0912217 PMC2943767

[34] O'Rorke MA, Ellison MV, Murray LJ, Moran M, James J, et al (2012) Human papillomavirus related head and neck cancer survival: A systematic review and meta-analysis. Oral Oncol 10.1016/j.oraloncology.2012.06.019 22841677

[35] Moore CB, Bergstralh DT, Duncan JA, Lei Y, Morrison TE, et al (2008) NLRX1 is a regulator of mitochondrial antiviral immunity. Nature 451: 573–577. 18200010 10.1038/nature06501

[36] Woods KV, El-Naggar A, Clayman GL, Grimm EA (1998) Variable expression of cytokines in human head and neck squamous cell carcinoma cell lines and consistent expression in surgical specimens. Cancer Res 58: 3132–3141. 9679981

[37] Pries R, Wollenberg B (2006) Cytokines in head and neck cancer. Cytokine Growth Factor Rev 17: 141–146. 16540364 10.1016/j.cytogfr.2006.02.001

[38] Brailo V, Vucicevic-Boras V, Lukac J, Biocina-Lukenda D, Zilic-Alajbeg I, et al (2011) Salivary and serum interleukin 1 beta, interleukin 6 and tumor necrosis factor alpha in patients with leukoplakia and oral cancer. Med Oral Patol Oral Cir Bucal 10.4317/medoral.17323 PMC3448188 21743397

[39] White E (2012) Deconvoluting the context-dependent role for autophagy in cancer. Nat Rev Cancer 12: 401–410. 22534666 10.1038/nrc3262 PMC3664381

[40] Collisson EA, Cho RJ, Gray JW (2012) What are we learning from the cancer genome? Nat Rev Clin Oncol 10.1038/nrclinonc.2012.159 PMC4169265 22965149

