# Supplementary figures and images for: Identification of Mutations in the PYRIN-Containing NLR Genes (NLRP) in Head and Neck Squamous Cell Carcinoma
Source: PLoS One. 2014 Jan 21;9(1):e85619. doi: 10.1371/journal.pone.0085619 (PMC3897487; doi:10.1371/journal.pone.0085619)

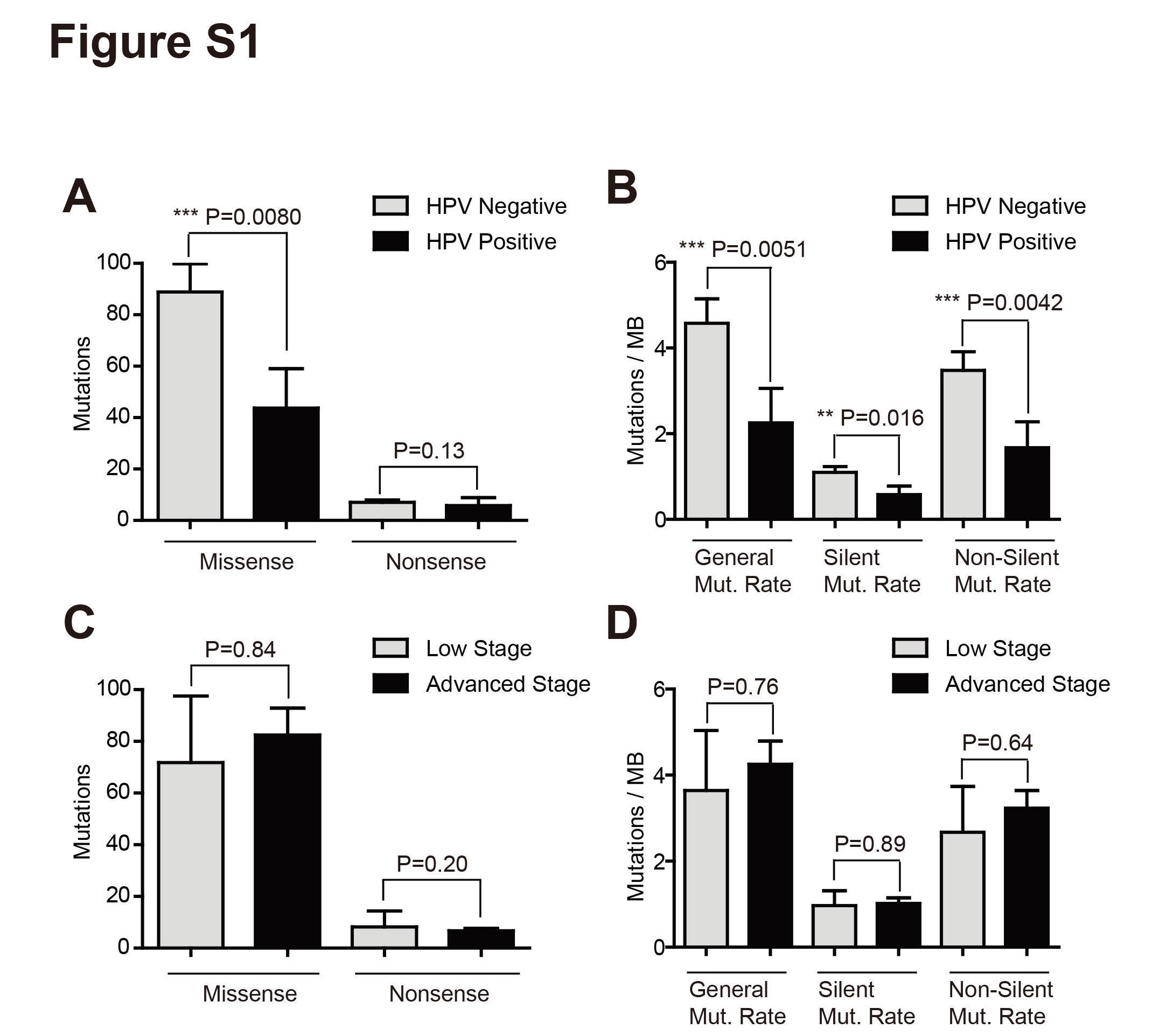

Supplement: Figure S1 — Mutation rates comparisons. (A) Numbers of missense and nonsense mutations were compared between patients with or without HPV infection. (B) Mutation rates were compared between patients with or without HPV infection. (C) Numbers of missense and nonsense mutations were compared between patients with low stage or advanced stage SCC. (D) Mutation rates were compared between patients with low stage or advanced stage SCC. P value less than 0.05 was considered significant. (TIF) [file pone.0085619.s001.tif]

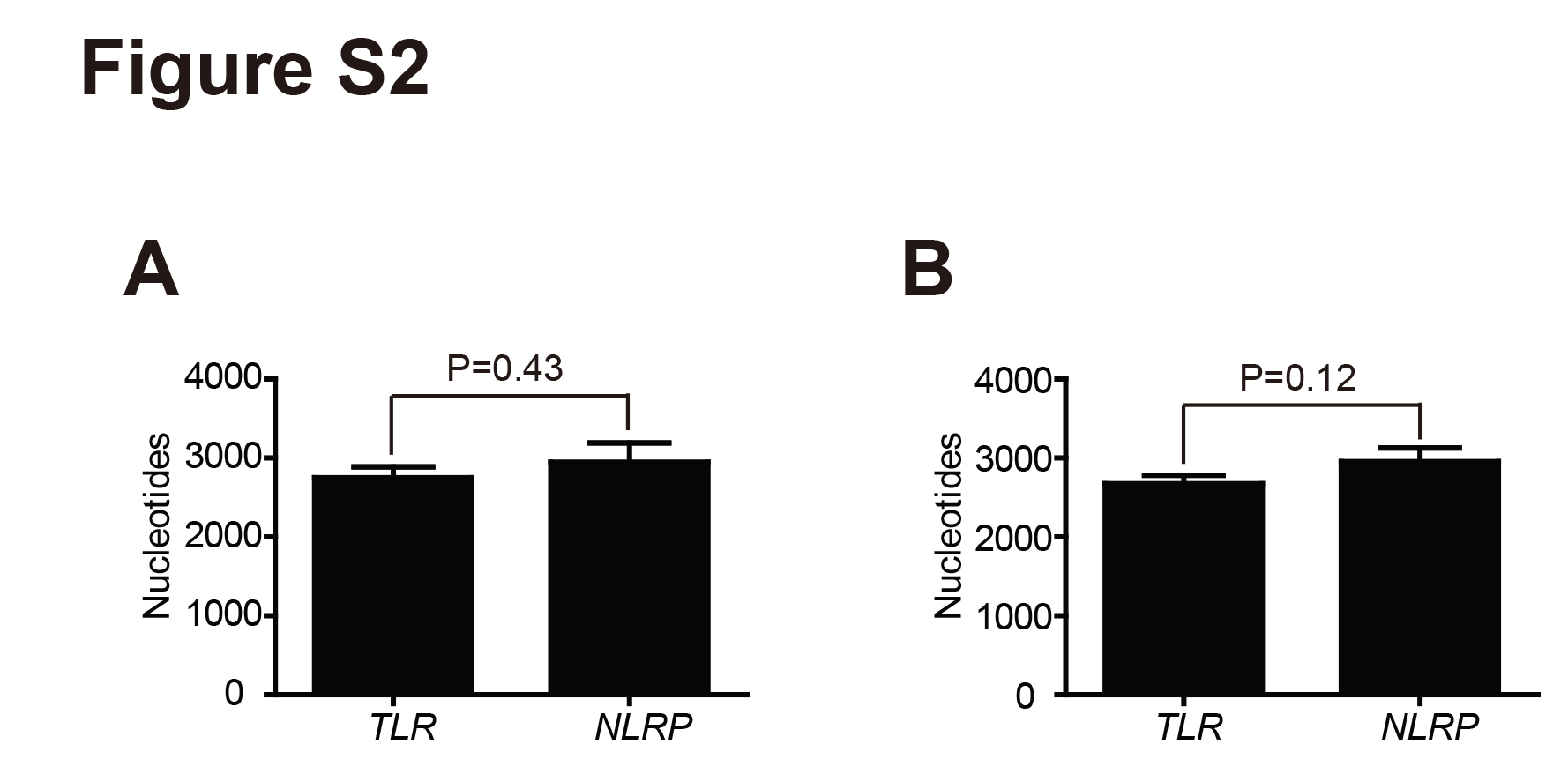

Supplement: Figure S2 — Coding region lengths comparisons. (A) The coding region lengths between selected members of the TLR and NLRP gene families, which were mutated in our cohort, were compared by Mann-Whitney U test. (B) The coding region lengths between the total members of the TLR and NLRP gene families were compared by Mann-Whitney U test. P value of less than 0.05 was considered significant. (TIF) [file pone.0085619.s002.tif]
