# Supplementary material for: Identification of Mutations in the PYRIN-Containing NLR Genes (NLRP) in Head and Neck Squamous Cell Carcinoma
Source: PLoS One. 2014 Jan 21;9(1):e85619. doi: 10.1371/journal.pone.0085619 (PMC3897487; doi:10.1371/journal.pone.0085619)
Supplement: Table S2 — Mutations of the TLR genes were not enriched in FOM HNSCC. Contingency table comparisons were made by Fisher's Exact test to investigate whether mutations of the TLR gene family were more frequently seen in HNSCC arising FOM. P value of less than 0.1 was considered significant. (DOCX) [file pone.0085619.s004.docx]

**Table S2. Mutations in *TLR* genes were not enriched in FOM HNSCC.**

|  | *TLR* Mut. | w/o *TLR* mut. | P value |
| --- | --- | --- | --- |
| FOM | 2 | 6 |  |
| Non-FOM  (all other OC locations) | 2 | 21 | 0.27 |
| FOM | 2 | 6 |  |
| Non-FOM  (all other H&N locations) | 5 | 49 | 0.22 |
